# ADAR1 Regulates Alternative Splicing Through an RNA Editing-Independent Mechanism

**DOI:** 10.3390/ijms27093952

**Published:** 2026-04-29

**Authors:** Eduardo A. Sagredo, Victor Karlström, Alejandro Blanco, Paloma Moraga, Matias Vergara, Aino I. Jarvelin, Neus Visa, Katherine Marcelain, Alfredo Castello, Ricardo Armisén

**Affiliations:** 1 Department of Molecular Biosciences, The Wenner-Gren Institute, Stockholm University, Svante Arrhenius 20C, 106 91 Stockholm, Sweden; victor.karlstrom@gmail.com (V.K.); neus.visa@su.se (N.V.); 2 Science for Life Laboratory, Department of Microbiology, Tumor and Cell Biology, Karolinska Institutet, 171 65 Solna, Sweden; 3 Centro de Genética y Genómica, Instituto de Ciencias e Innovación en Medicina, Facultad de Medicina, Clínica Alemana Universidad del Desarrollo, Santiago 7550000, Chile; ablanco@udd.cl (A.B.); matvergara19@gmail.com (M.V.); 4 Biomedical Neuroscience Institute, Faculty of Medicine, University of Chile, Santiago 8380453, Chile; p.moragaschaaf@gmail.com; 5 FONDAP Center for Geroscience, Brain Health and Metabolism (GERO), Santiago 7550000, Chile; 6 Department of Biochemistry, University of Oxford, South Parks Road, Oxford OX1 3QU, UKalfredo.castello@glasgow.ac.uk (A.C.); 7 Departamento de Oncología Básico Clínica, Facultad de Medicina, Universidad de Chile, Santiago 8380453, Chile; kmarcelain@uchile.cl; 8 Centro para la Prevención y el Control del Cáncer (ACCDiS), Universidad de Chile, Santiago 8380453, Chile; 9 MRC-University of Glasgow Centre for Virus Research, The University of Glasgow, Glasgow G61 1QH, UK

**Keywords:** ADAR1, alternative splicing, RNA editing, proteomics, ACIN1, spliceosome, epitranscriptomics

## Abstract

Dysregulation of the RNA-editing enzyme ADAR1 is associated with human diseases, including cancer, but its RNA-editing-independent roles in alternative splicing remain largely unexplored. Comprehending these molecular mechanisms is paramount, as they may unveil novel therapeutic targets. This study elucidates how the ADAR1p110 isoform influences alternative splicing independently of its canonical editing activity. Employing RNA-sequencing, proteomic analysis of ADAR1p110 interactors, and functional assays with wild-type and mutant ADAR1 in diverse human cell lines (including cancer models), we investigated its impact on the splicing landscape. Our findings indicate that ADAR1p110 interacts with pivotal spliceosome components and auxiliary splicing regulators. Notably, ADAR1 extensively modulates alternative-splicing events, with most of these alterations occurring independently of its RNA-editing activity and often its RNA-binding capacity. Furthermore, ADAR1 alters the isoform expression of other splicing factors (e.g., ACIN1), suggesting an indirect regulatory mechanism. Importantly, this splicing reprogramming affects genes that govern therapeutic response, positioning the ADAR-splicing axis as a potential driver of drug resistance. By revealing this predominantly editing-independent mechanism, we expand the understanding of ADAR1’s non-canonical functions and identify a new avenue for therapeutic intervention in cancer.

## 1. Introduction

The adenosine deaminase acting on RNA (ADAR) enzymes catalyzes adenosine-to-inosine (A-to-I) RNA editing, one of the most prevalent RNA modifications in mammals [1,2,3]. Inosine is recognized as guanosine by the cellular machinery, which can have profound effects on RNA structure and translational fidelity [4,5,6]. The ADAR protein family consists of three members (ADAR1-3), two of which are catalytically active (ADAR1 and ADAR2) [3]. All ADAR enzymes contain two to three double-stranded RNA-binding domains and a deamination domain [7]. ADAR1 is ubiquitously expressed and has two isoforms, ADAR1p110, which is primarily nuclear and nucleolar, while ADAR1p150 is induced by interferon and can shuttle between the nucleus and the cytoplasm [8,9]. Changes in ADAR1 expression and activity are associated with various diseases, including amyotrophic lateral sclerosis [10], systemic lupus erythematosus [11], and several types of cancer [10]. Recent data suggest a broad role of ADAR proteins in cell fate, showing that they can modify mRNA splicing sites [11] and alternative polyadenylation sites [12] and affect miRNA processing/maturation [13,14] and RNA stability [15,16,17] by altering canonical RNA–RNA and RNA–protein interactions [18]. Several studies have established how RNA editing may influence alternative splicing by modifying splicing regulatory elements or by altering dsRNA structures. These alterations may affect the affinity of the splicing machinery and trans-acting factors for those regions, thereby altering splicing efficiency [11,19,20,21,22]. One of the most well-characterized RNA-editing-dependent splicing events involves the glutamate receptor subunit 2 (GRIA2) transcript [23], where a single recoding site of the GRIA2 pre-mRNA, in the amino acid 607 (known as Q/R site), is required for the excision of the intron 11, regulating the processing and proper mRNA cytoplasmic localization [24,25,26]. Moreover, ADAR2 can edit its transcript, generating a new splice site that results in a transcript variant encoding a truncated, non-functional protein [25,27]. As virtually all mRNAs are spliced and a vast majority undergo RNA editing co-transcriptionally, it has been proposed that these two processes can mutually influence each other. Indeed, transcriptomic analyses revealed that alterations in ADAR1 and ADAR2 expression significantly affect exon usage and alternative splicing in animal and cellular models [11,19,28,29]. In contrast, pharmacological inhibition of the spliceosome has been shown to influence ADAR editing at sites that compromise introns or other RNA structures that can enhance RNA editing due to secondary structure [19,29].

ADAR1-knockout mice die at early embryonic stages due to widespread apoptosis [30,31,32]. This phenotype can be partially rescued by reintroducing catalytically inactive ADAR1 mutants, indicating that A-to-I editing is not the only role of ADAR1 in RNA metabolism [33,34]. In agreement, it has been shown that ADAR can interact with other RBPs and influence their activity, further reinforcing the idea of an editing-independent function [18]. In the early days of genomic studies, Solomon et al. (2013) [11] intersected alternative splicing and RNA editing analyses following ADAR1 knockdown, showing that RNA editing can regulate the generation of mRNA variants. However, the extensive changes in alternative splicing observed upon ADAR1 knockdown could not be fully attributed to editing, and, indeed, only a few editing sites were detected in the vicinity of near-regulatory splicing sequences. Similarly, Licht et al. [21] demonstrated that ADAR2 RNA binding could influence splicing efficiency in an editing-independent manner for specific ADAR2 target minigene constructs. In agreement, Kapoor et al. (2020) [20] recently showed that Adar −/− mouse-derived tissues display extensive editing-independent splicing changes.

In this work, we investigated the role of ADAR1 in alternative splicing across four non-related transgenic cell lines, focusing on the connections between RNA editing and alternative splicing, and showed that most splicing events upon ADAR1 knockdown or overexpression are not RNA-editing-dependent. We also established the ADAR1p110 protein–protein interactome, showing interactions with several key spliceosome co-factors. Furthermore, using two ADAR1p110-inducible cell lines, we found that most ADAR1-related splicing events do not involve RNA editing near the affected splicing junctions. Finally, we show that overexpression of ADAR1p110 alters the exon usage of splicing factor isoforms in the cell, suggesting that ADAR1 exerts indirect effects on splicing. These results highlight a complex regulatory network of splicing mediated by ADAR1 that includes direct RNA editing and indirect effects on the splicing machinery.

## 2. Results

### 2.1. Editing of Splicing Motifs Does Not Correlate with Alterations in Exon Usage upon ADAR1 Expression

**Figure 1:**
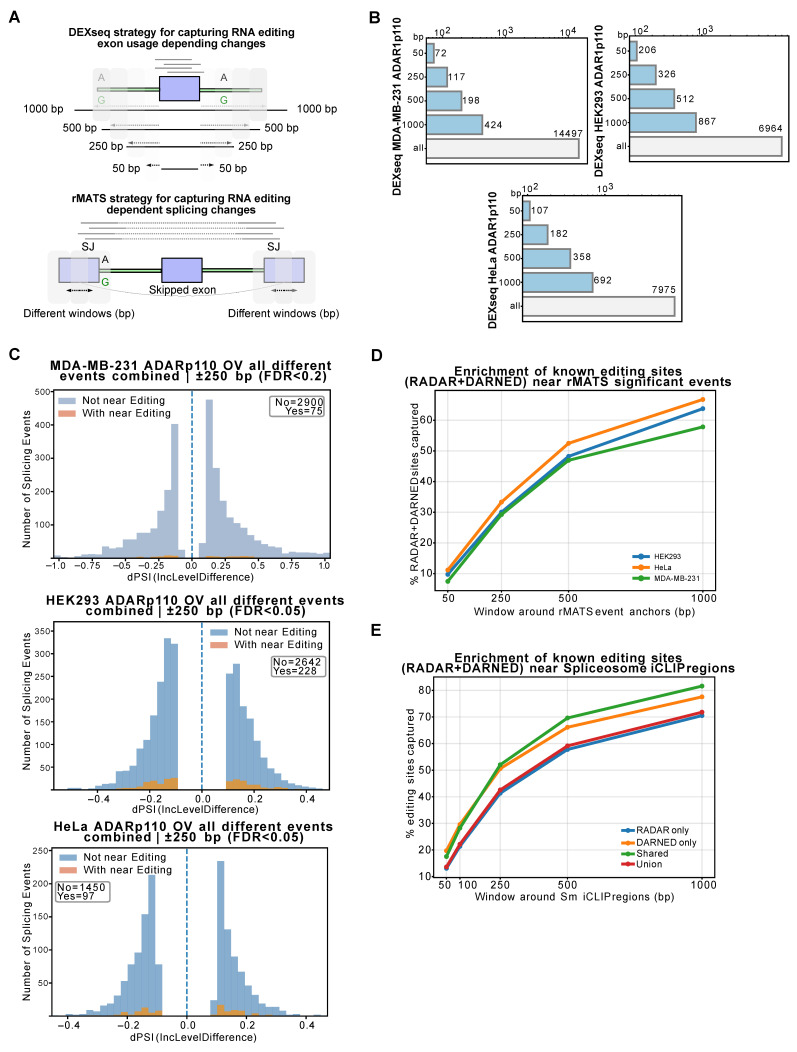
**ADAR1 manipulation produces significant splicing changes that are not entirely attributable to its A-to-I activity**. (**A**) Differential exon usage (DEU) and rMATS splicing strategies used to correlate feature changes with RNA-editing events. (**B**) Number of DEUs with addressable editing sites in the compromised exon position with an extended boundary of 50 bp, 250, 500, or 1000 bp, using their variant-calling information: MDA-MB-231 ADAR1p110-overexpressing cells, HEK293 ADAR1p110 OV cells and HeLa ADAR1p110 OV cells (FDR < 0.05). (**C**) Binned intersection of rMATS significant changes with their variant calling using a 250 bp window for the three different cell lines used in this study (FDR < 0.2 for MDA-MB-231 cells; FDR < 0.05 for HEK293 and HeLa cells). (**D**) Percentage of editing sites present in RADAR and DARNED databases and their association, using different overlap windows for the three different cell lines used in this study (**E**) Percentage of editing sites present in RADAR and DARNED databases and their association with the spliceosome CLIP positions based on Briese et al.’s study (2019) [35] and their intersection using an extended boundary of 50 bp, 250 bp, 500 bp, or 1000 bp.

To explore ADAR’s role in splicing regulation, we created HeLa and HEK293 Flp-In TRex cells that express a GFP-tagged ADAR1p110 protein in response to doxycycline (Figure S1). We conducted RNA-seq experiments followed by differential exon usage (DEXseq) and differential alternative splicing analyses (rMATS), comparing cells overexpressing ADAR1p110 with those overexpressing GFP. Additionally, we included a previously published RNA-seq dataset of MDA-MB-231 cells overexpressing ADAR1p110. To correlate our data with RNA-editing events, we performed an RNA-editing variant calling using the unfiltered RNA variant events to maximize the detection of any potential RNA-editing site and further intersect those with two different strategies. The first was based on the exonic region identified by our DEXseq analysis (DEU), and the second on the splicing junctions annotated by rMATS. Briefly, from all differential features, we searched RNA-editing sites in their vicinity using different windows ranging from 50 to 1000 base pairs (bp), in both directions, including those RNA-editing sites annotated in the RADAR and DARNED databases, to reach any previously described editing sites that may not have been identified by our variant calling (Figure 1A). Our DEXseq contrast, even when using a larger overlap window (1000 bp), revealed that fewer than 15% (12.4% for the HEK293 ADAR1p110 OV cells) of the differentially expressed exons contained RNA-editing sites in their coordinates across the different models (Figure 1B). In addition to this, using a 250 bp window for our splicing junction approach, based on rMATS contrasted with our variant calling, displayed a limited number of RNA-editing-dependent changes (75 out of 2975 intersections for the MDA-MB-231 ADAR1p110-transduced cells; 228 out of 2870 intersections in our HEK293-overexpressing cells; and 97 out of 1547 intersections in our HeLa-overexpressing model) (Figure 1C). As expected, the overlap between differentially spliced events and edited bases increased as the window size increased (Supplementary Figure S2A). However, despite using a larger window (1000 bp), only ~30% of differentially spliced events overlapped with editing sites (Supplementary Figure S2A). We further contrasted the differential-splicing event against the RADAR and DARNED databases, showing that no more than 60% of the splicing changes displayed previously known RNA-editing events, even when a 1000 bp window was used to correlate them (Figure 1D). When non-significant splicing changes, or those significant splicing events with a limited magnitude (dPSI < ±0.1), were contrasted with the same databases, they displayed comparable accumulation curves across the 50–1000 bp range across all three cell lines (Figure S2B), suggesting that the proximity between editing sites and splice sites reflected a constitutive genomic architecture rather than a feature selectively enriched at differentially spliced events. To reinforce these results, we compiled the spliceosome-binding sites reported by previous iCLIP-published datasets. We tested the binding footprints on the RNA against known RNA-editing events. The overlap between the spliceosome iCLIP and the editing databases RADAR and DARNED was only 3.33%, yielding 96,896 editing sites from a total of 2,909,674 sites in RADAR and DARNED. However, the overlap increased to 85.4% when a 1000 bp window was used. Altogether, these complementary analyses revealed an apparent lack of correlation between ADAR1-mediated RNA edits and alternative-splicing events (Figure 1E).

### 2.2. The Protein–Protein Interactome of ADAR1p110 Includes Many Splicing Factors

**Figure 2:**
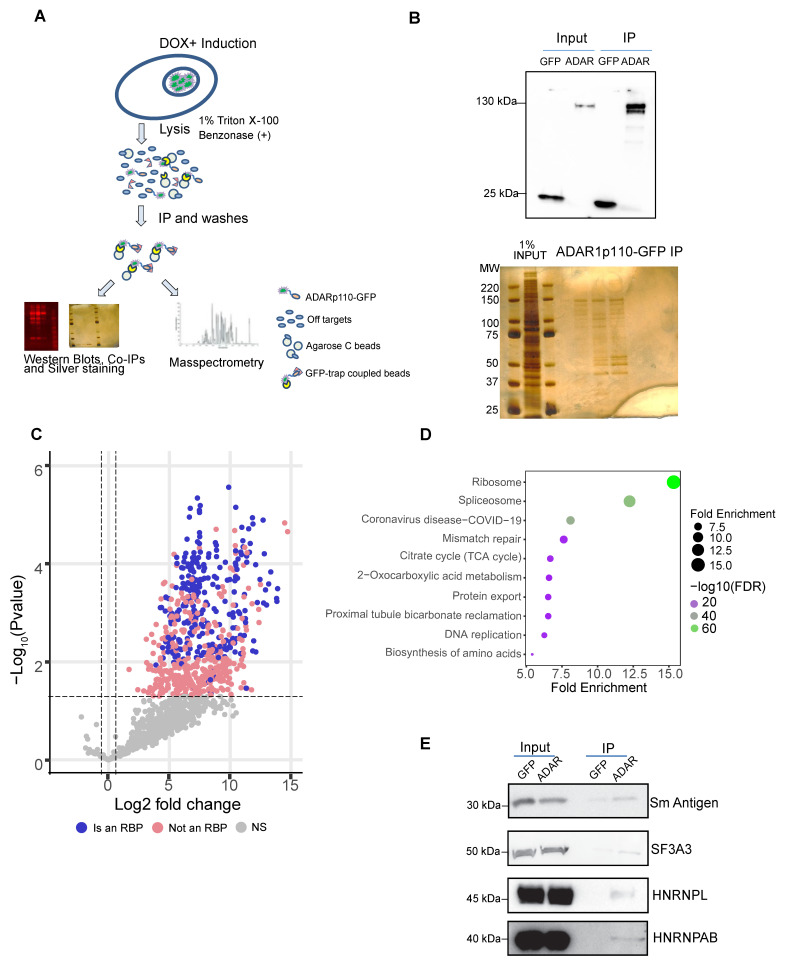
**HR-MS reveals that ADAR1p110 interacts with splicing machinery**. (**A**) Model of work for HR-MS to capture ADAR1-p110 protein interactors. (**B**) Representative immunoprecipitation for HeLa ADAR1p110 and HeLa GFP cells, and silver staining’s PAGE gel for HeLa ADAR1p110-induced cells, showing inputs and their respective pull-downs. (**C**) Volcano plots showing significant ADAR protein–protein interactors, highlighting significant (pink) and known RNA-binding protein (RBP) (blue) interactors. Dashed lines depict significant -Log10 *p*-value (FDR) (y-axis > 1.75) and Log2 fold change over GFP (x-axis > 1). (**D**) KEGG pathway enrichment for Q1 and Q2 IBAQ-ranked ADAR1p110 interaction partners. (**E**) co-IP validations in HeLa cells overexpressing GFP or ADAR1p110, showing inputs and IP elutions for Sm Antigen, SF3A3, HNRNPL, and HNRNPAB.

Classical biochemistry assays have associated ADAR1 with RNA-binding proteins involved in splicing, such as HNRNPL, NONO, and SFPQ [36]. To study all the protein partners of ADAR1p110, we used our HeLa Flp-In TRex cells that express a GFP-tagged ADAR1p110 and performed co-immunoprecipitation with GFP-trap beads under non-denaturing conditions to identify further their protein–protein interactions by label-free quantitative proteomics (Figure 2A). GFP-expressing control cells were used to exclude contaminants, and the pull-down was verified by Western blot and silver-stained SDS-PAGE (Figure 2B). The analysis revealed 432 significantly enriched proteins in ADAR1p110-GFP. As immunoprecipitation was performed in native conditions, these proteins reflected direct and indirect protein-bridged interactors. Notably, 270 of the interactors were annotated as RBPs, 163 of which harbored classical RNA-binding domains (RBDs), and 107 were non-canonical [37,38] (Figure 2C and Supplementary Table S1). To focus on the most prominent (stoichiometric) interactors, we selected the proteins with a higher eluate signal (q50 quantile of the IBAQ signal). We performed pathway enrichment analyses using the KEGG, Reactome, and STRING databases. We found a significant enrichment of proteins involved in ‘ribosome’ (FDR: 1.0 × 10^−80^), ‘Coronavirus disease-COVID-19’ (FDR: 1.1 × 10^−59^), ‘spliceosome’ (FDR: 1.2 × 10^−37^), and ‘mRNA surveillance’ (FDR: 6.8 × 10^−6^), among others, categories related to RNA metabolism (Figure 2D and Supplementary Table S2). We observed a striking enrichment of members of the Sm complex and U2 snRNP complex in the ADAR1p110 interactome (SF3A3, SF3B2, SF3B3, SNRPB2, SNRPA1, SNRPD3, SNRPE, and SNRPG) [39] (Supplementary Table S2). We also identified splicing regulators, including HNRNPL, DHX15, SFPQ, and STRBP [40]. We validated several of the observed interactions by ADAR1 immunoprecipitation followed by Western blot, showing that the Sm antigen, the splicing factor SF3A3, and two heteronuclear proteins, HNRNPL and HNRNPA/B, were enriched, reinforcing the significance of our results (Figure 2E). These results imply that ADAR1 may also regulate splicing through interaction with the spliceosome and several of its cofactors.

### 2.3. Deconvoluting the Role of ADAR1p110 in Splicing

**Figure 3:**
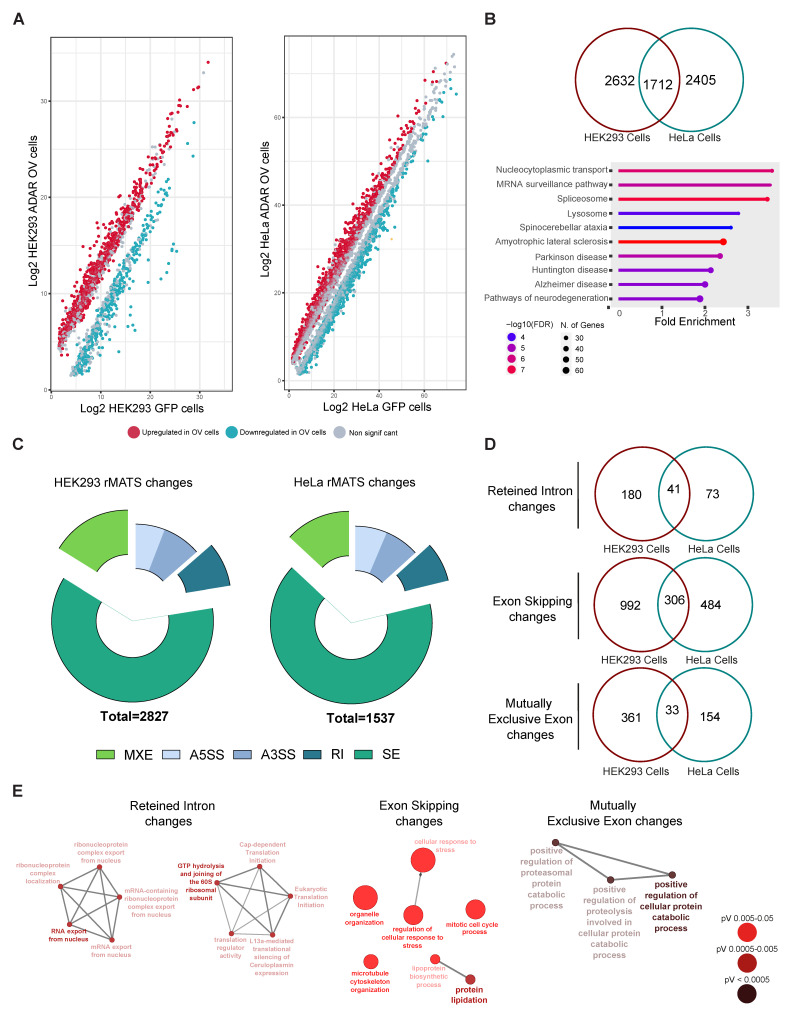
**ADAR overexpression in HeLa and HEK293 models produces significant splicing changes**. (**A**) Differential exon usage (DEU) counts in HEK293 (left) and HeLa ADAR1p110-overexpressing cells, showing those exons significantly overrepresented (included) in red and those excluded after ADAR1p110 overexpression (in green). (**B**) The Venn diagram shows the overlap—at the transcript level—of significant DEU changes in HEK293 and HeLa cells (upper), and the significant biological processes associated with the shared transcripts with significant DEU counts in both cell lines. (**C**) Pie charts showing the significant splicing change types after rMATS analysis in HEK293 ADAR1p110-overexpressing (left) and HeLa ADAR1p110-overexpressing (right) cells. (**D**) Venn diagrams showing the overlap in the different splicing event changes between HEK293 and HeLa ADAR1p110-overexpressing cells. (**E**) Gene enrichment analysis (Biological process) for those splicing events shared between HEK293 and HeLa ADAR1p110 cells.

Our results indicate an editing-independent function of ADAR1 in splicing, consistent with the presence of many splicing factors in ADAR’s interactome. Using DEXseq from both ADAR1p110-GFP HeLa and HEK293 Flp-in TREx cells, we observed that 4207 and 4235 exons had decreased usage in HeLa and HEK293, while 3751 and 2720 exons showed higher usage than GFP-induced control cells (Figure 3A and Supplementary Table S3). To characterize transcripts with changes in exon usage, we compared transcript-level results across both lines, identifying 1712 transcripts that were differentially spliced in an ADAR-dependent manner in both cell lines. These genes were involved in the ‘RNA processing’ (FDR: 1.4 × 10^−30^), ‘protein localization’ (protein localization to organelles (FDR: 4.8 × 10^−24^)), and ‘viral processing’ (FDR: 3.2 × 10^−22^) GO terms, among others. These terms were highly enriched for RBPs and splicing-related factors (Figure 3B and Supplementary Table S4).

Furthermore, we utilized rMATS to analyze splicing events, an event-based software that circumvents the limitations of differential exon usage based on exonic counts. Using this tool, we detected 1537 significant alternative-splicing changes in HeLa cells and 2827 in HEK293 cells upon ADAR1p110-GFP overexpression, compared with GFP-induced cells (Figure 3C). Most of the changes were related to exon skipping (SE), representing 65.7% and 61.4% in HeLa and HEK293 cells, respectively, upon ADAR1p110 overexpression. The following most abundant splicing events were mutually excluded exon changes (MXEs) (13% and 16.1%, respectively), intron retention (RI) (7.9% and 8.9%, respectively), and alternative 5′ and 3′ splice site usage (13.3% and 13.6%, respectively) (Figure 3D and Supplementary Tables S5 and S6), indicating that ADAR1p110 exerts alternative-splicing changes on many transcripts across different cell types. However, many of the observed transcripts exhibiting splicing changes did not overlap across cell lines, suggesting that ADAR1-dependent splicing regulation varies strongly with cellular condition and cell type (Figure 3D).

GO analysis revealed that transcripts with intron retention changes in both cell lines were enriched for RNA splicing and RNA processing (FDR: 0.042) and cytoplasmic translation (FDR: 0.042). The transcripts that exhibited significant exon-skipping changes were enriched in ‘protein lipidation,’ ‘organelle organization’ (organelle assembly and microtubule cytoskeleton organization; FDR: 0.009), and DNA damage repair-related GO terms (i.e., ‘mitotic cell cycle’; FDR: 0.009). Conversely, significant mutually exclusive exon changes were enriched in protein catabolic-related processes (‘pos. reg of cellular protein metabolic processes’; FDR: 0.02) (Figure 3E). Finally, we found that several splicing factors, including THOC1, ACIN1, TIA1, SRSF6, HNRNPD, DDX46, and U2AF1, exhibited significant alterations in their splicing patterns upon ADAR1p110 induction. Furthermore, genes with central roles in the cell cycle and DNA damage response, such as BRCA1, PAX6, MCM3, MKI67, and BCLAF1, also displayed differential splicing in both cell lines.

Our next goal was to validate the ADAR1p110-induced changes in alternative splicing, testing their dependence on RNA editing and the RNA-binding activity of ADAR1p110. To do so, we transfected HEK293 cells with an editing-deficient ADAR1 mutant (ADAR1-DeaD) and an RNA-binding-deficient mutant (ADAR1-EAA) (Figure S3A). Testing differential exon usage identified by our DEXseq analyses, including PTPR, HNRNPC, LIG1, NME, and SRSF1. Using splice-site-specific primers, we tested for exon inclusion and exclusion. Our results revealed that ADAR1 WT overexpression and its mutants induced similar splicing changes, suggesting that ADAR1p110 can affect splicing in an RNA-binding and activity-independent manner (Figure S3B).

### 2.4. Elucidating the Editing Dependence of ADAR1-Mediated Changes in Differential Splicing

To characterize the editing-independent role of ADAR1p110 in alternative splicing, we performed an A-to-I variant calling on the whole transcriptome of HeLa and HEK293 T-REx cells. We intersected those sites with the altered splicing junctions in our data. As expected, most of the detected editing sites (~90%) occurred in Alu-elements situated in non-coding genomic regions (Supplementary Table S4), and less than 0.3% of detected edited sites were found in coding regions (Figure S4A, left panel). In addition, we performed a GO analysis on the significant edited sites shared between HEK293 and HeLa cells at the gene level (1598 genes), showing enrichment for ‘cell cycle’ (FDR: 0.0001), ‘DNA replication’ (FDR: 0.002), ’Fanconi anemia pathway’ (FDR: 0.001), ‘mismatch repair’ (FDR: 0.04), and ‘homologous recombination’ (FDR: 0.02) GO terms, among others (Figure S4A, right panel).

**Figure 4:**
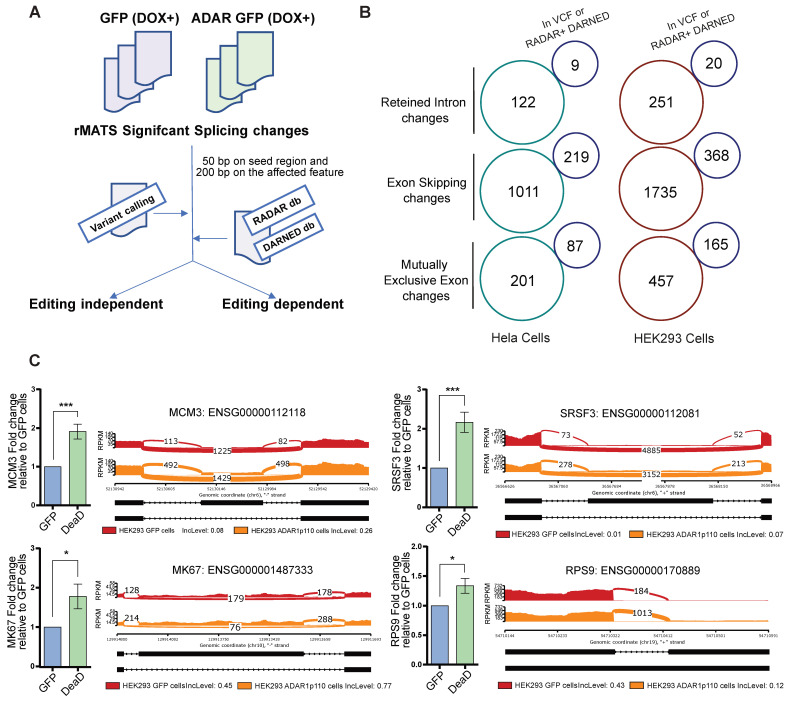
**ADAR1p110 overexpression in HeLa and HEK293 cells produces significant splicing changes that are not entirely associated with their A-to-I activity**. (**A**) Workflow to identify and recall splicing variants that are affected by editing events and those recalled as editing-independent events. Briefly, rMATS significant sites were intersected with the variant-calling files and the RADAR + DARNED databases, using a strategy that comprises the affected feature and seed region, from the affected splicing change (for further details, see the Materials and Methods). (**B**) Identification of RNA-editing-dependent and RNA-independent events in HeLa and HEK293 cells, showing the different splicing change types in the data and the number of events (in blue) that can be described as editing-dependent at the gene level. (**C**) qRT-PCR validation for rMATS splicing events recalled as editing-independent events after overexpressing HEK293 cells with GFP or the DeaD ADAR1p110 plasmid. In addition, sashimi plots are shown for those targets validated by qRT-PCR. Two-tailed Student’s T-test was used to calculate differences between samples in E (n = 3) (* = *p* < 0.05, *** = *p* < 0.001).

We designed a workflow to intersect differential splicing events with RNA-editing variants in our data, and to identify RNA-editing sites included in different RNA-editing databases, considering the splice junction and the intronic region near any significant splicing change (Figure 4A and Materials and Methods). Interestingly, less than 5% of the splicing changes in our data intersected with RNA-editing variants called in the cell lines (Figure S3B,C, left panels), suggesting that most changes are unlikely to be directly related to RNA editing. From the different splicing changes, mutually exclusive exon (MXE) changes had the highest number of edited sites in their sequence. In contrast, the 5′ splice site, 3′ splice site, and intron retention changes had a lower frequency of RNA-editing sites in their coordinates (Figure 4B and Supplementary Tables S5 and S6), where a small fraction intersected in the RADAR, DARNED and/or Reditools databases (Figure 4B and Figure S4B,C, right panels). Furthermore, we selected several transcripts exhibiting alternative splicing in the absence of RNA editing for validation by qRT-PCR. Those targets were related to DNA damage response and alternative splicing, including MCM3, RPS9, MKI67, and SRSF3. Strikingly, we observed that overexpression of the ADAR1-DeaD mutant led to significant changes in the alternative splicing of these transcripts, supporting that the alteration of their splicing is editing-independent (Figure 4C).

### 2.5. ADAR1p110 Regulates and Contributes to Transcriptome Variability Affecting mRNAs That Encode for Splicing Cofactors

Our results showed that many ADAR1-regulated alternative-splicing events appeared to be editing-independent. To test this phenomenon, we co-transfected the different ADAR1p110 mutants with a splicing reporter consisting of an intron-containing luciferase cDNA. We observed a notable decrease in luciferase signal upon overexpression of ADAR1p110-DeaD and ADAR1p110-EAA in HEK293 cells (Figure S5A). These results agree with Kapoor et al.’s findings (2020) [20] and suggest that non-catalytically active ADAR1p110 influences the splicing of the reporter in both an editing-independent and RNA-binding-independent manner. We had two hypotheses: (1) ADAR regulates splicing factor function through direct protein–protein interactions by changing their RNA-binding properties or stability; (2) the activity of ADAR on mRNAs induces changes in splicing that alter the configuration of splicing factors, leading to differential effects.

**Figure 5:**
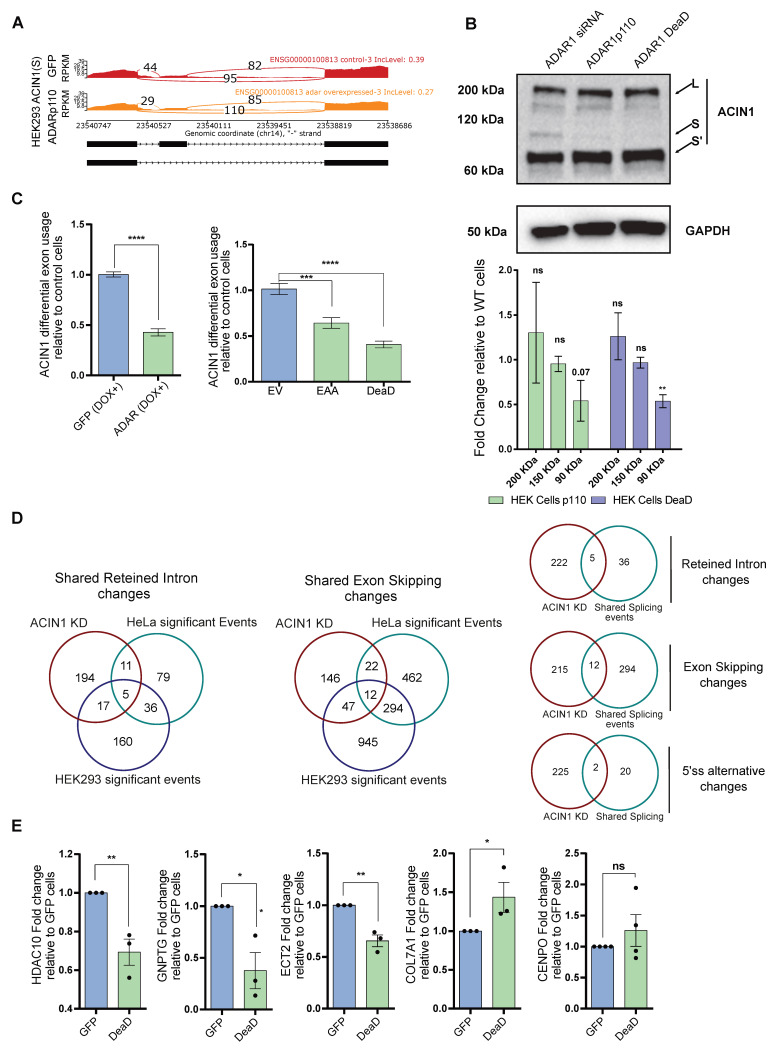
**ADAR manipulation produces essential changes in splicing activity and the alternative splicing of ACIN1**. (**A**) Sashimi plots showing ACIN short variants (S and S’) on HEK293 ADAR1p110 and GFP OV cells, showing their different exon inclusion patterns. (**B**) Representative Western blot showing ACIN1 isoforms after ADAR1 knockdown (siRNA), ADAR1p110 overexpression, and ADAR1 DeaD overexpression in HEK293 cells, with their respective quantification for those bands associated with the larger isoform (200 kDa, 150 kDa, and 90 kDa). (**C**) ADAR overexpression leads to a significant change in ACIN1 exon usage, independent of editing activity. (**D**) Intersection between splicing events produced by ACIN1, after ACIN1 knockdown, and the significant splicing events found in either HeLa or HEK293 ADAR1p110-overexpressing cells, displaying the intersection for all the different splicing event types at the gene level. (**E**) qRT-PCR measuring ACIN1-splicing events after ADAR1 DeaD overexpression, compared with HEK293 cells transfected with GFP, showing significant changes in the splicing pattern of previously validated ACIN1 targets. Two-tailed T-test was used to calculate significant differences between samples (* = *p* < 0.05, ** = *p* < 0.01, *** = *p* < 0.001, and **** = *p* < 0.0001) in (**B**,**C**,**E**) (n = 3).

One splicing co-factor affected by ADAR overexpression was ACIN1, a protein component of the apoptosis- and splicing-associated protein complex (ASAP) that interacts with the exon junction complex (EJC) [41,42]. The transcript of ACIN1 consistently showed editing-independent alternative splicing and expression changes in both our HeLa and HEK293 ADAR1p110-overexpressing cells (Figure 5A and Figure S5B). Indeed, the overexpression of both DeaD and EAA ADAR1p110 in HEK293 cells led to a significant change in the ACIN1 isoform pattern, both at the protein level (Figure 5B) and mRNA level (Figure 5C), suggesting that ADAR1 could indirectly modify the splicing process by affecting splicing configuration, which we expanded to other splicing factors (Figure S3B). To evaluate whether the second hypothesis is correct, we intersected publicly available transcriptome data from studies investigating the effects of alternative splicing following ACIN1 knockdown [42,43] with our data obtained after overexpression of ADAR1p110 (Figure 5D). Interestingly, some targets were shared and affected the same mRNA-splicing variants in both the ACIN1 dataset and our experiments. Furthermore, we performed qRT-PCR in the same splicing regions associated with ACIN1 using the DeaD ADAR1p110 variant. Notably, the overexpression of DeaD ADAR1p110 can partially reconstitute the effects of ACIN1 (Figure 5E). Together, these results reinforce the idea that ADAR1 can indirectly regulate the alternative splicing of specific transcripts by modulating the splicing of transcripts encoding splicing factors, revealing an intricate role for ADAR1 in regulating alternative splicing and downstream phenotypes.

## 3. Discussion

In this work, we provided new evidence supporting the role of ADAR1p110 in regulating alternative splicing. It has previously been suggested that ADAR1 interacts with the spliceosome and that ADAR1p110 can be detected in the same fraction as U1 and U2 spliceosome complexes [44]. Previous reports have shown that the editing function can be regulated by splicing co-factors such as SRSF9 and DDX15 [45,46] and that RNA immunoprecipitation experiments with HNRNP proteins reveal interactions with Alu regions and other features strongly associated with ADAR1-binding sites [36,47]. Moreover, some cofactors of the spliceosome, such as U2AF2, can be detected near editing sites [45], suggesting that these factors could interact with or displace ADAR1, thereby modifying ADAR1 activity. Our ADAR1p110 protein–protein interactome experiment showed that many ADAR1p110 interactors are indeed RNA-binding proteins involved in RNA metabolism and alternative splicing, which is consistent with other recent ADAR1 proteomic studies [46,48], strengthening the argument that ADAR1p110 could act as a splicing regulator.

We investigated transcriptome-wide changes in alternative splicing following manipulation of ADAR1 expression and found thousands of changes in each of the three cell lines tested. Our data indicate that more than half of the observed differential exon usage in MDA-MB-231, HEK293, and HeLa ADAR1-manipulated cells does not occur near an editing site. Even when using a screening window of 1000 bp and relaxed criteria for editing determination, we found no correlation between splice sites and editing, in agreement with the previous literature [49]. In further support of the editing-independent regulation of alternative splicing by ADAR1, we observed a low overlap of known edited sites when intersecting their positions with the published iCLIP data for the spliceosome [35]. Together, these findings suggest that a significant proportion of the differential splicing events observed following ADAR1 dysregulation are driven by its editing-independent functions.

Upon ADAR1p110-DeaD overexpression, we observed significant changes in the luciferase reporter plasmid, with ADAR1p110-EAA also inhibiting the reporter’s splicing signal compared with the WT protein. Complementary to this, previous works have also investigated the overall role of deficient ADAR enzymes in mice, observing that the RNA-binding-deficient Adar1 protein or Adar1 knockout induces a substantial impact on the splicing landscape across different mouse tissues [21,22], in agreement with our results.

After overexpressing ADAR1p110-GFP in HEK293 and HeLa cells, we observed that the differentially spliced transcripts were enriched for DNA repair, mRNA surveillance pathways, splicing/spliceosome, and other related processes. Interestingly, many of the observed changes in differential splicing generated transcripts annotated as coding for novel protein isoforms. This phenomenon was previously demonstrated for the ADAR-regulated differential splicing of the CCD15 and RELL2 transcripts [22]. Although the extent of the ADAR1p110-mediated perturbations to alternative splicing remains to be investigated, it may influence mRNA decay or protein isoform production, favoring mRNA variants targeted for degradation via mechanisms such as nonsense-mediated decay (NMD) or producing novel protein isoforms. In line with this, one potential explanation for ADAR1-regulated splicing changes that occur without local RNA editing is the modulation of “poison exons” within essential splicing factors [50]. This possibility exists alongside other established models, such as ADAR1’s direct physical interaction with core spliceosome components or its ability to alter the expression levels of key splicing regulators. In this context, it is conceivable that high ADAR1 levels might shift the splicing balance of master factors like SRSF2, SRSF6, or ACIN1 toward isoforms containing premature termination codons. Such a mechanism could potentially trigger NDM of these transcripts, leading to a systemic depletion of the splicing machinery. While this hypothetical alternative splicing–NMD axis remains to be formally dissected, it offers a plausible indirect pathway that could complement direct protein–protein interactions to drive the widespread transcriptomic remodeling and “splicing addiction” observed in our cell models. These results could also add an extra layer of complexity to pathological contexts linked to ADAR1, where this protein is significantly overexpressed and could influence transcript stability and isoform generation, alterations implicated in many disorders and types of cancers [49,51]. This study provides a mechanistic rationale for ADAR1’s role in drug resistance. We propose that high ADAR1 levels, common in aggressive breast tumors, co-opt the splicing machinery not only through direct interaction but by modulating the splicing of splicing factors themselves (e.g., ACIN1). This ‘splicing of splicers’ effect amplifies the regulatory reach of ADAR1, specifically altering isoforms of genes critical for cell survival and DNA repair (e.g., BRCA1 and BCLAF1) under therapeutic pressure. This establishes a pro-survival splicing program that drives resistance, effectively creating a ‘splicing addiction’, where tumor cells become dependent on ADAR1’s non-canonical function to maintain the expression of therapeutic resistance isoforms.

ADAR1p110-GFP overexpression affected the splicing and expression of ACIN1, a core component of the ASAP complex involved in alternative splicing [41], nonsense-mediated decay [43], apoptosis, and chromatin condensation [52]. After intersecting the observed differential splicing data in our HEK293 and HeLa cells with differential splicing data available for ACIN1 knockdown [44], we determined that a certain proportion of splicing events overlapped between the datasets, indicating the same type of differential splicing at the same gene coordinates. Suggesting that the observed splicing perturbations could be due to the effect that ADAR1p110 exerts on ACIN1, rather than to any direct relationship between ADAR1p110 and those transcripts, results that we further validated by qRT-PCR.

RNA editing occurs co-transcriptionally, and most sites are present in intronic regions [28,53]; a critical challenge in these studies is correlating editing with alternative splicing and sufficient read coverage to limit false positives. As our RNA-seq data are based on a poly-A-enriched strategy, which limits intronic regions, we supplemented our editing data with the extensive data in the Reditools, RADAR, and DARNED databases to increase sensitivity in capturing splicing events associated with RNA editing. However, even with very relaxed parameters (1000 bp) in each direction from the differentially used exons, less than 30% contained edited sites near splicing junctions. Seeing that ADAR1p110 also affects the splicing of multiple other splicing factors and interacts with them at a protein–protein level, the full extent of ADAR1’s involvement in alternative splicing should be investigated systematically to determine these effects in cis or trans.

## 4. Materials and Methods

### 4.1. Cell Culture

HeLa and HEK293 cells were cultured under standard conditions at 37 °C in a humidified incubator containing 5% CO_2_. HeLa ADAR1p110 Flp-In T-REx and GFP Flp-In T-REx cells were generated, subcloning the ADAR1p110 cds into the pCDNA5-FRT-cGFP plasmid to further integrate the construct using the Flp-In system following the manufacturer’s instructions (Thermo Fisher Scientific, Carsland, CA, USA).

### 4.2. Immunoblotting and Co-Immunoprecipitation

HeLa ADAR1p110 Flp-In T-REx cells were lysed (one 10 cm dish at 80% (800,000 cells) confluency per immunoprecipitation experiment) in 1 mL of lysis buffer (10 mM Tris/Cl pH 7.5; 150 mM NaCl; 0.5 mM EDTA; 0.5% Triton X) supplemented with protease inhibitors (Halt, Thermo Fisher Scientific, Carsland, CA, USA) and 250 units of Benzonase (Sigma Aldrich, Darmstadt, Germany), and placed on ice for 30 min. At the same time, it was extensively shredded using a G15 syringe. Cell lysates were centrifuged at 20,000 rpm for 10 min at 4 °C, and the supernatant was transferred to a low-binding pre-cooled 1.5 mL tube. An amount of 40 μL of a pre-washed control bead (Pierce) (washed three times in cold lysis buffer supplemented with 1 mM DTT and proteinase inhibitors) slurry was added to the lysate and incubated for 30 min at 4 °C on a rotating platform. Lysates were spun down at 2500 rpm for 2 min, and the supernatant was transferred to a new pre-cooled 1.5 mL low-binding tube, incubated with 40 μL of pre-washed GFP-trap beads (washed three times in cold lysis buffer supplemented with 1 mM DTT (Thermo Fisher Scientific, Carsland, CA, USA) and protease inhibitors) for 4 h under gentle rotation at 4 °C. The complexed beads were spun down at 4 °C at 2500 rpm for 2 min, and the supernatant was discarded to further wash them three times with lysis buffer (supplemented with protease inhibitors). Finally, the complexed beads were resuspended in 40 μL of buffer. For the co-immunoprecipitation experiments, 10 μL of the complexed beads was boiled with NuPAGE Buffer (4X) (Thermo Fisher Scientific, Carsland, USA) and 3 μL of 1 M DTT, following the manufacturer’s recommendations. Western blotting was performed as described previously in Sagredo et al.’s study (2018) [54]. Silver staining was performed according to the manufacturer’s protocol and recommendations for the SilverQuest Silver Staining Kit (Thermo Fisher Scientific, Carsland, CA, USA). For mass spectrometry, the beads were eluted using a pH elution as indicated in the manufacturer’s protocol (GFP-trap_A; Chromotek, Munich, Germany). Mass spectrometry was performed as previously described by Avolio et al. (2018) [55].

### 4.3. Reporter Assay and Transfections

Reporter assays were performed according to Armisén et al. (2011) [56]. Briefly, HeLa cells were transfected with 1 µg of the CMV-LUC2CP/ARE plasmid (Addgene #62857), 0.5 µg of the Renilla plasmid (pRL-Renilla, Promega, Madison, WI, USA), and 1 µg of the different ADAR1 isoform plasmids. ADAR1p110 deaminase inactive (DeaD) and the ADAR1p110 dsRBD mutant (EAA) were cloned into a pCDNA3.1(+) backbone derived from ADAR1p110 cDNA. Dual-Luciferase measurements were performed using the Dual-Glo Luciferase system kit (Promega, Madison, WI, USA) and the Cytation 3 Multi-Mode Reader (BioTek Instruments, Santa Clara, CA, USA). The Renilla signal was used to normalize prior luciferase comparisons between the different samples. Plasmid and siRNA transfections were carried out as described by Sagredo et al. (2020) [16]. Briefly, the different plasmids used in this study were transfected using Lipofectamine 3000 (Thermo Fisher Scientific, Carsland, CA, USA) in six-well plates at 80% confluency, according to the manufacturer’s instructions. For siRNA control (Cell Signaling, Boston, MA, USA) or ADAR1 (Thermo Fisher Scientific, Carsland, CA, USA), siRNAs were transfected at a final concentration of 20 nM and incubated for 48 h for further experiments. For plasmid transfection, 2.5 µg of the different plasmids was transfected, and 16 h after transfection, the cells were grown in fresh media and harvested 48 h after transfection.

### 4.4. RNA-Seq Experiments and Workflow

RNA integrity and RNA sequencing were performed as described previously by Sagredo et al. (2020) [16]. Fastq files were aligned using STAR (two-step mode) against hg19, and transcripts were counted with the HT-seq software (Version 2.0.5). Differential exon usage (DEU) analysis for the different cell lines and datasets included in this study was performed using the DEXSeq [57] tool v1.36.0 (DEXseq_count.py script) to further perform the differential DEU analysis, as recommended by the software developers. Features with FDR < 0.05 or FDR < 0.2 for the MDA-MB-231 cells were included in the analysis. Differential splicing events were called and analyzed using the rMATS-turbo v4.1.2 software [58,59], comparing control samples with ADAR1-overexpressing cells. Splice junction definitions from the UCSC goldenPath repository were utilized for further differential splicing analysis, using FDR < 0.05. All generated data were processed in R (3.6.1) or the Unix command line. Datasets from MDA-MB-231 cells were analyzed by Sagredo et al. (2020) [16] (SRA accession number: SRP200634).

### 4.5. RNA Editing and Splicing Intersection Analysis

Similar to Kapoor et al. (2020) [20], we intersected the differential exon usage data analyzed with the DEXseq tool. We scanned the called editing sites in 50 bp, 250 bp, 500 bp, and 1000 bp windows on both sides of the exon/intron boundary. In addition, reference sites present in the RADAR and DARNED databases were also included [53,60]. This analysis was performed for significant differential exons, including both included/excluded features. RADAR and DARNED events were annotated and intersected with the spliceosome iCLIP from Briese et al.’s study (2019) [35], E-MTAB-8182, using bedtools intersect v2.26.0, and the same window-scanning strategy was used to intersect both datasets.

rMATS splicing events were called in default mode (--b1 {bams_controls} --b2 {bams_oe} --gtf hg19.ensGene.gtf -t paired --readLength 100 --nthread 12 --od ./output_files/ --tmp ./output_files/tmp --libType fr-firststrand). For further analysis, the files counted only the reads that used cross-splice junctions. The splicing events with fewer than 20 reads between the sum of counts with and without in each replicate, including the exon/intron/splice event, were removed. The remaining events were filtered by FDR ≤ 0.05, with a difference between control and OE groups of |IncLevelDifference| ≥ 0.1. Where IncLevelDifference is the difference in the PSI (percent spliced in) values of the control vs. OE treatment groups. The filtered set of splicing events was then annotated with external_gene_name using the biomaRt package v2.54.0 from Bioconductor. For the significant splicing changes, we selected a set of windows involving the splicing event. The window considered 50 bp from the splice junction into the body of the exons and 200 bp into the introns of the event, except for RI events, where 50 bp was considered for both directions of the splice junctions. This set of windows was created for each significant splicing event and intersected with the set of edited sites. The edited sites were filtered for total depth ≥ 5 (considering all samples, 6 per cell type) and retained those observed in at least two replicates or in the RADAR and/or DARNED databases [53,60]. Then, both sets, splicing events and edited sites, were intersected with bedtools v2.26.0 using bedtools intersect. Also, the splicing events were annotated with RADAR + DARNED, obtained from REDIportal [61], using the same “windows set” strategy to account for known editions that intersect the splice-site windows. If any splicing event intersected with the sample’s editions or a known edition from RADAR + DARNED, the event was tagged as editing-dependent; otherwise, it was tagged as editing-independent. rMATS significant changes were additionally annotated and intersected with the ADAR1 iCLIP data from the studies by Chen et al. (2015) (GSE63709) [14] and Bahn et al. (2015) [12] (GSE55363) and the irCLASH data from Song et al.’s study (2020) [62] (GSE136327).

### 4.6. Quantitative RT-PCR

Total RNA was extracted using the Mammalian Total RNA Isolation Kit following the manufacturer’s instructions (SIGMA-Aldrich, Darmstadt, Germany). This included Turbo DNase (Thermo Fisher Scientific, Carsland, CA, USA) treatment, following the manufacturer’s instructions. An amount of 500 ng of RNA was reverse-transcribed using an AffinityScript qRT-PCR cDNA Synthesis Kit (Agilent Technologies Inc., Santa Clara, CA, USA) and diluted 4 times. qRT-PCR was performed using specific primers (for further details, please check Table S7) and the KAPA SYBR Green master mix (Kapa Biosystems, Wilmington, DE, USA). The reactions were performed on the MIC qPCR machine using the following thermal profile: 95 °C for 15 s, 58 °C for 15 s, and 72 °C for 15 s, with 40 cycles, including no-template controls. Expression values were calculated using the ΔΔCt method and expressed as the fold change relative to control samples. ACTNB was used as a housekeeping gene. In addition, RESS-qPCR for AZIN1 and MDM2 targets was performed as described by Crews et al. (2015) [63]. Primers were used to validate the DEU data, targeting a specific exon and normalizing to GAPDH. For rMATS validations, two primer sets were used to measure splicing changes, amplifying the affected exon and a constant region of the targeted transcript.

### 4.7. Site-Level A-to-G(I) Editing Comparisons in HeLa and HEK293 FLP T-REx Cells

Variant calling, annotations, and filtering were performed as described previously in the studies by Sagredo et al. (2020) [16] and Widmark et al. (2022) [64]. Only addressable A-to-G(I) variants were used; Fisher’s exact test was used to compare the treatments, and a confidence level of 0.05 was used as a cut-off for further analysis. All generated data were processed in R (3.6.1).

### 4.8. Gene Ontology and Pathway Enrichment Analysis

To determine the relationship between ADAR1 and splicing regulation, a gene ontology analysis was carried out using the Cytoscape [65] v3.7.1 software and the ClueGO [66] v2.5.4 plugin, or the ShinyGO [67] 0.76.3 web application. For the ClueGO analysis, a gene list was submitted to this software using the Biological Process database (v06/01/2022) for further comparisons. Only statistically significant groups were displayed, using a Bonferroni step-down multiple comparison post hoc test. Corrected *p* < 0.05 was considered statistically significant. For the protein–protein enrichment analysis, the STRING [68] database webpage was used, using the complete list (Uniprot IDs) of significant targets (FDR < 0.05, compared with GFP samples).

## 5. Conclusions

This study shows that ADAR1, especially ADAR1p110, regulates alternative splicing. ADAR1p110 interacts with spliceosomal components and auxiliary splicing factors and modulates the isoform landscape of other splicing regulators. This affects the transcriptome indirectly. This discovery reveals a new function of ADAR1, orchestrating a complex network that governs splicing outcomes beyond direct editing of splice sites. Future investigations into ADAR1’s editing-independent regulatory axis will help understand its diverse functions in cellular homeostasis and its contributions to human diseases, such as cancer. ADAR1 expression is often dysregulated in cancer, and its impact on splicing could be a key pathological characteristic.

## Data Availability

The data associated with this article are available in its online Supplementary Materials. The RNA-sequencing data are available under the PRJNA1444515 accession ID. The raw proteomic files are available in MassIVE under the accession ID MSV000101300.

## Supplementary Materials

The following supporting information can be downloaded at https://www.mdpi.com/article/10.3390/ijms27093952/s1.

## Abbreviations

The following abbreviations are used in this manuscript:

A-to-I	Adenosine-to-Inosine
ACIN1	Apoptotic Chromatin Condensation Inducer 1
ADAR	Adenosine Deaminase Acting on RNA
ASAP	Apoptosis- and Splicing-Associated Protein
bp	Base Pair
cDNA	Complementary
DNA	DeaD Deaminase Inactive
DEU	Differential Exon Usage
dsRBD	Double-Stranded RNA-Binding Domain
EAA	RNA-Binding Deficient Mutant
EJC	Exon Junction Complex
FDR	False Discovery Rate
GFP	Green Fluorescent Protein
GO	Gene Ontology
GRIA2	Glutamate Ionotropic Receptor AMPA Type Subunit 2
iCLIP	Individual-Nucleotide Resolution Cross-Linking and Immunoprecipitation
MXE	Mutually Exclusive Exons
NMD	Nonsense-Mediated Decay
OE	Overexpression
PSI	Percent Spliced In
qRT-PCR	Quantitative Real-Time Polymerase Chain Reaction
RADAR	Rigorously Annotated Database of A-to-I RNA Editing
RBP	RNA-Binding Protein
RI	Intron Retention
SE	Skipped Exon
shRNA	Short-Hairpin RNA
siRNA	Small Interfering RNA
snRNP	Small Nuclear Ribonucleoprotein
WT	Wild Type
