# Supplementary material for: ADAR1 Regulates Alternative Splicing Through an RNA Editing-Independent Mechanism
Source: Int J Mol Sci. 2026 Apr 29;27(9):3952. doi: 10.3390/ijms27093952 (PMC13164374; doi:10.3390/ijms27093952)
Supplement: Supplementary file 1 [file ijms-27-03952-s001.zip › Supplementary Materials Captions.pdf]

Supplementary Figure S1. Characterization of the ADAR1p110 Flp-In TREx system. **A**) schema of Flp-In TREx ADAR1p110 GFP doxycycline-inducible system. **B**) GFP fluorescence intensity signal produced in HeLa ADAR1p110-GFP cells after doxycycline induction, recorded during 24h. **C**) mRNA levels of ADAR1 after doxycycline induction on HEK293 and HeLa cells. **D**) RESS-qPCR for RNA editing targets (AZIN1 and MDM2) on HeLa and HEK293 ADAR1p110 after and before doxycycline induction. E) Confocal imaging showing ADAR1p110 localization, showing nuclear staining (DAPI) and ADAR1p110-GFP (FITC) (63X oil magnification). Two-tailed student's T-test was used to calculate differences between samples in C (n=3) (**=P<0.01, ***=P<0.001, ns: non-significant).

Supplementary Figure S2. ADAR1p110 overexpression produces significant splicing changes. **A**) Distribution of All significant splicing events on the three different models used in this study using different spanning windows, showing events containing RNA editing events in yellow. **B**) Enrichment percentage for those non-significant splicing changes and low dPSI significant changes across different spanning windows in all three models used in this work.

Supplementary Figure S3. ADAR1 manipulation produces significant exon usage changes on transcripts. **A**) ADAR mutants used for the different transfections, showing the different mutations present across ADAR1p110 domains. **B**) qPCR validations for differential exon usage not related to ADAR1 editing activity, showing significant changes after DeaD and EAA overexpression in HEK293 cells for PTPRS, HNRNPC, LIG1, NME and SRSF1. Two-tailed student's T-test was used to calculate significant differences between samples (ns= non-significant, *=P<0.05, **=P<0.01, ***=P<0.001) (n=3).

Supplementary Figure S4. Distribution of RNA editing and Splice Junctions in HeLa and HEK293 cells and their association. **A**) RNA editing characterization in HeLa and HEK293 cells after performing variant calling, showing the number of editing sites, their distribution on Alu regions and across the mRNA features. **B**) Pie charts show the overlap between high-confidence splice junctions and RNA editing sites (identified in this study or present in the REDIportal database). High-confidence splice junctions (top two quartiles) were extracted from STAR SJ.out.tab files from both HeLa and HEK293 cell lines, adding a 500 bp genomic window around each junction (+/- 250 bp) using bedtools slop (-b 250) and subsequently intersected with the variant calling results from each particular cell line and the REDIportal RNA editing sites**.**

Supplementary Figure S5. RNA editing splicing reporter on ADAR1p110 models and Sashimi plots for ACIN1(L) in ADAR1p110-GFP and GFP induced cells. **A**) Splicing reporter activity after ADAR1 mutant overexpression. Briefly, a split Luciferase plasmid was co-transfected with the different ADAR1p110 mutants, normalized using a renilla plasmid. **B**). Sashimi plots showing ACIN long variant on HEK293 and HeLa ADAR1p110 and GFP OV cells.

Supplementary Table S1. ADAR1p110-GFP protein-protein interactome hits, showing the different proteins associated with ADAR1p110 compared to GFP.

Supplementary Table S2. STRING and Reactome enrichment analysis for ADAR1p110-GFP protein-protein interactome.

Supplementary Table S3. Differential exon usage changes in HeLa and HEK293 cells after ADAR1p110 induction (FDR<0.1).

Supplementary Table S4. GO (BP) enrichment analysis for those transcripts with DEU changes in HeLa and HEK293 cells after ADAR1p110 induction.

Supplementary Table S5. rMATS detected changes in HeLa cells after ADAR1p110 induction, annotated using RADAR and DARNED, Variant calling, spliceosome iCLIP, and ADAR1 iCLIP available data.

Supplementary Table S6. rMATS detected changes in HEK293 cells after ADAR1p110 induction, annotated using RADAR and DARNED, Variant calling, spliceosome iCLIP, and ADAR1 iCLIP available data.

Supplementary Table S7. List of PCR primers.
