# Supplementary material for: ADAR1 Regulates Alternative Splicing Through an RNA Editing-Independent Mechanism
Source: Int J Mol Sci. 2026 Apr 29;27(9):3952. doi: 10.3390/ijms27093952 (PMC13164374; doi:10.3390/ijms27093952)

Supp. Figure 2

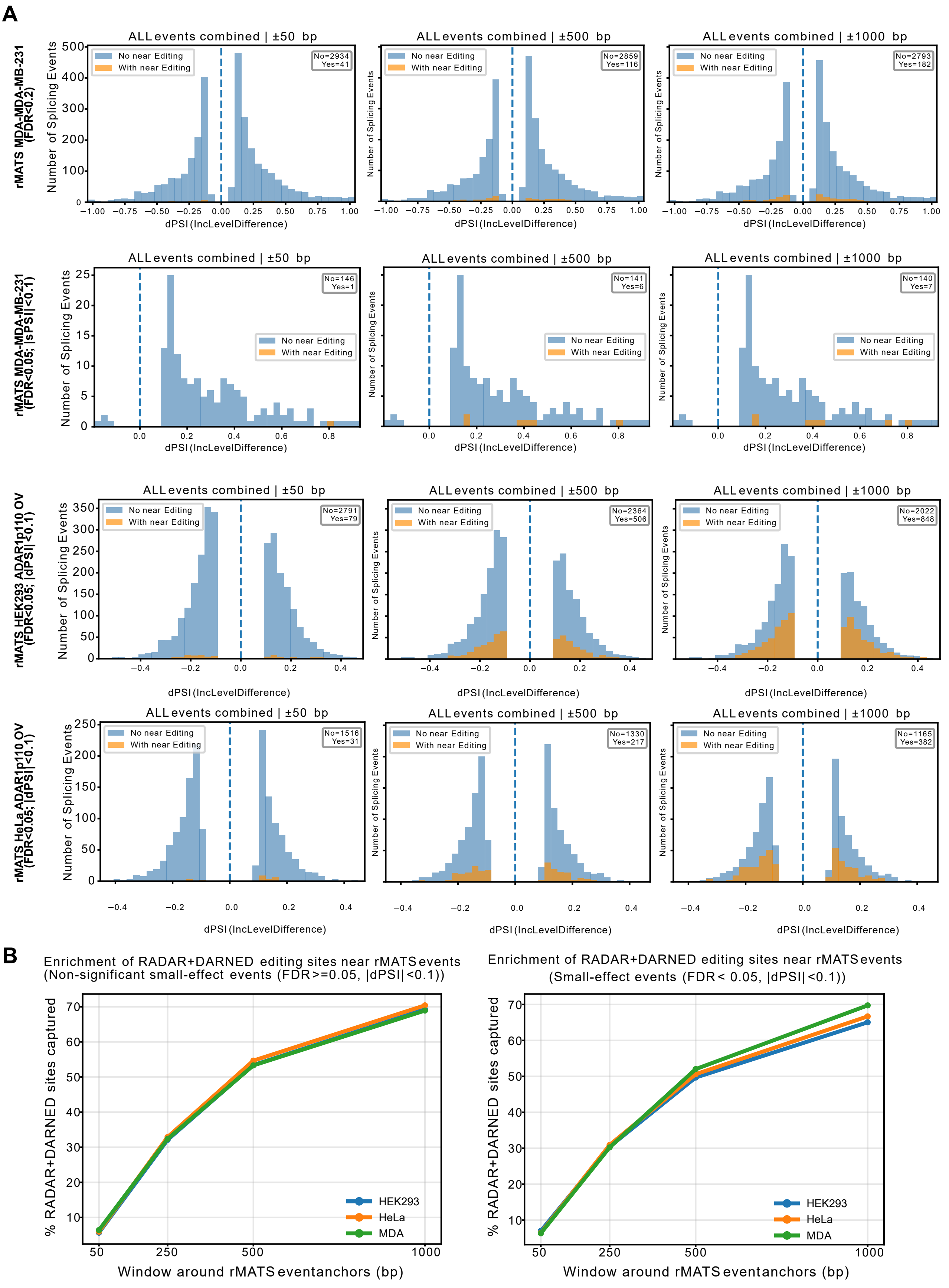

Supplement: Supplementary file 1 [file ijms-27-03952-s001.zip › Supp_fig2_April_v22026.pdf]
