# Supplementary figures and images for: ADAR1 Regulates Alternative Splicing Through an RNA Editing-Independent Mechanism
Source: Int J Mol Sci. 2026 Apr 29;27(9):3952. doi: 10.3390/ijms27093952 (PMC13164374; doi:10.3390/ijms27093952)

Supp. Figure 1

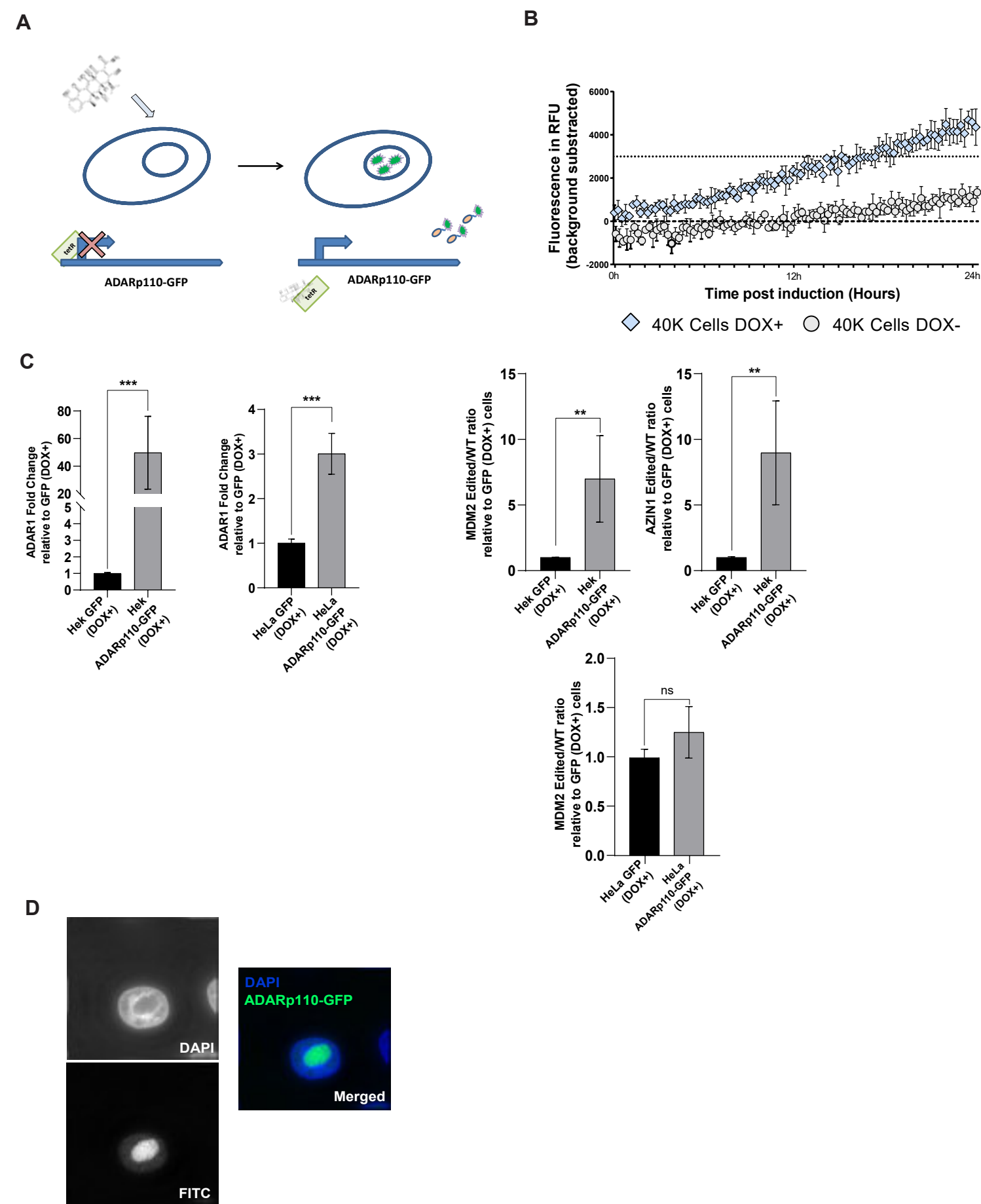

Supplement: Supplementary file 1 [file ijms-27-03952-s001.zip › Supp_fig1_April_v22026.pdf]

Supp. Figure 3

A

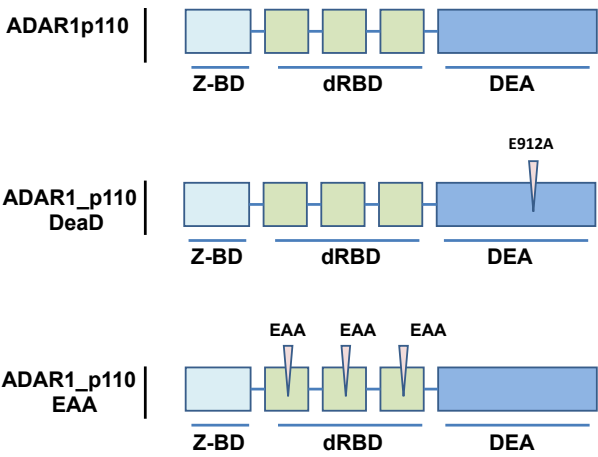

B

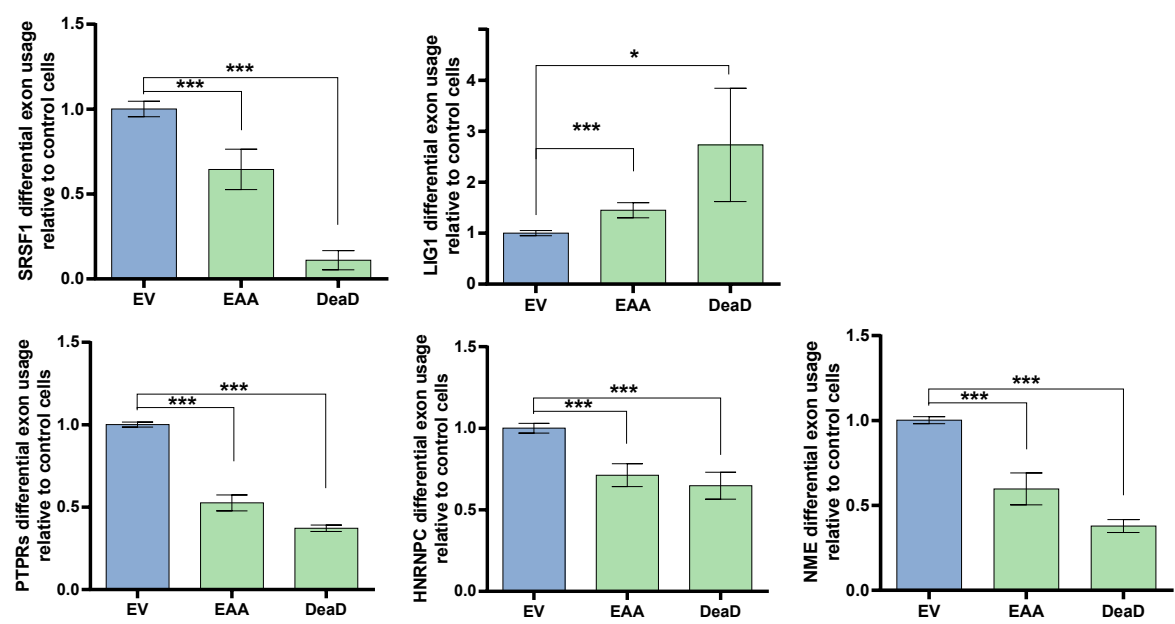

Supplement: Supplementary file 1 [file ijms-27-03952-s001.zip › Supp_fig3_April_v22026.pdf]

Supp. Figure 4

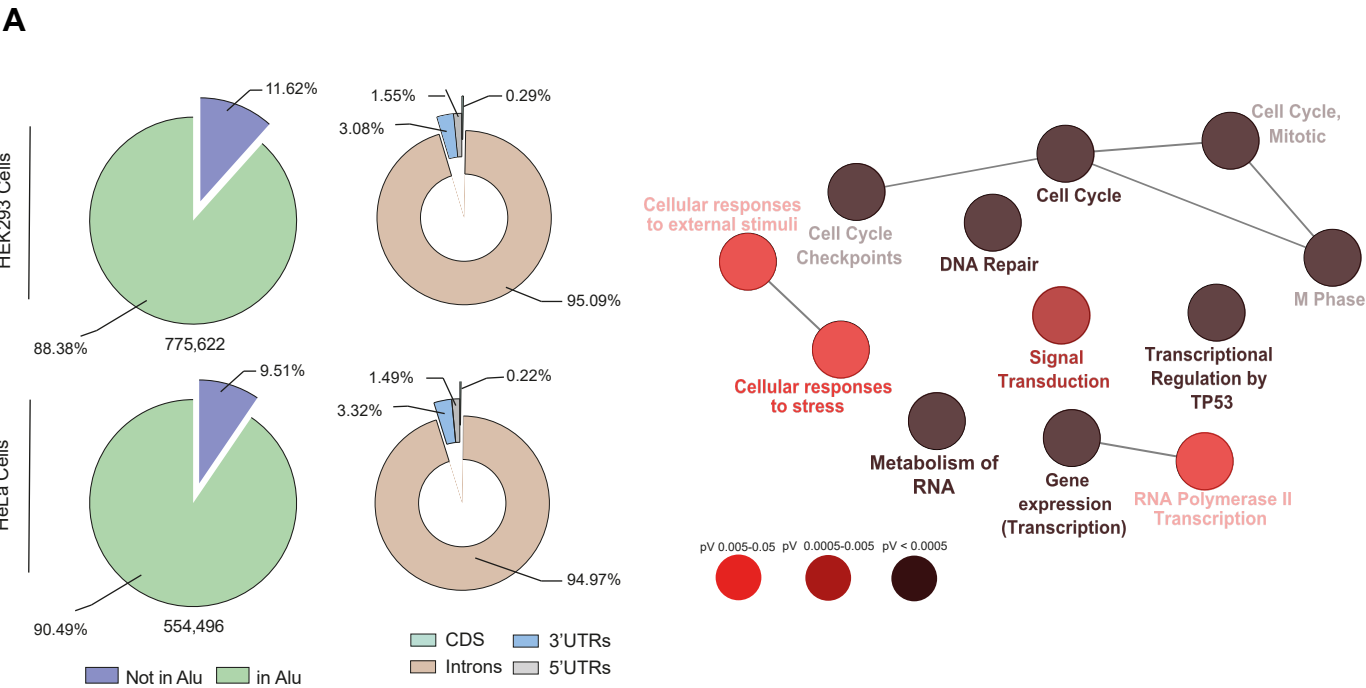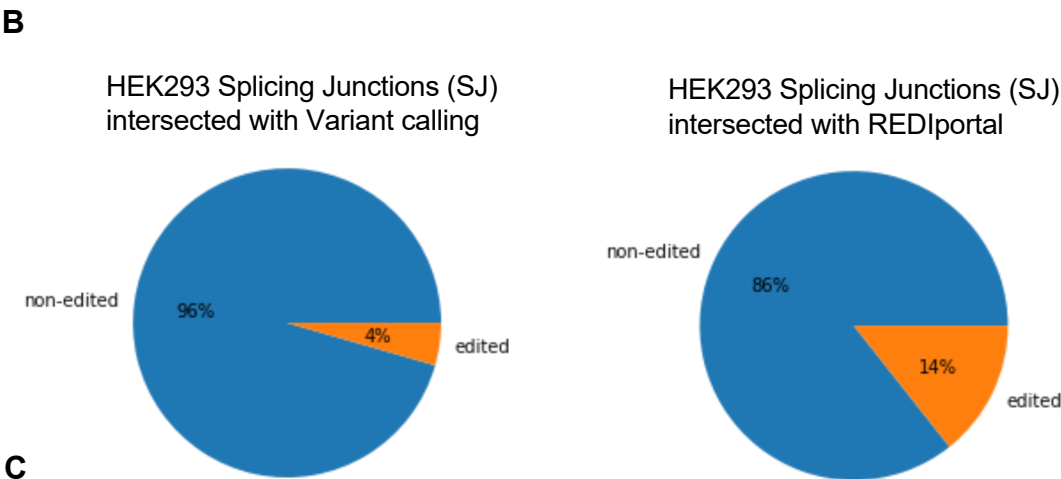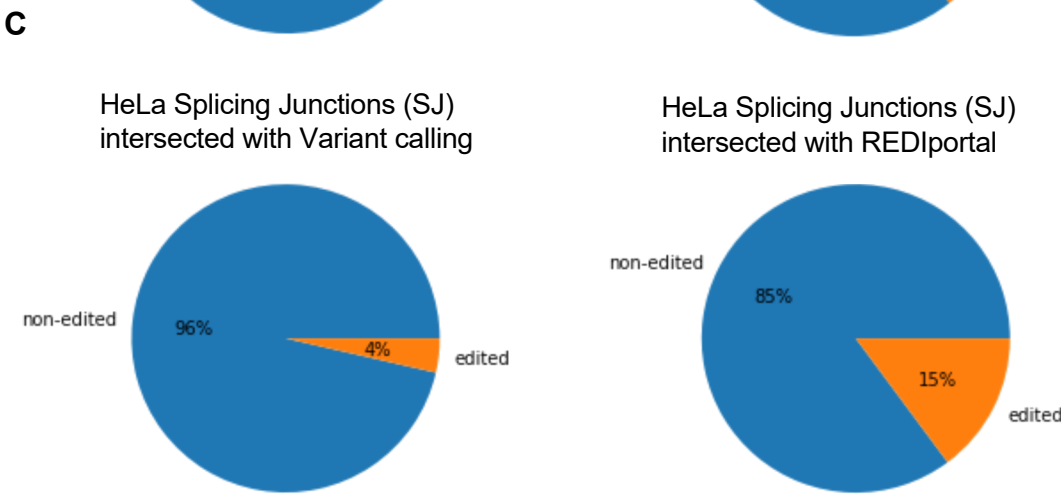

Supplement: Supplementary file 1 [file ijms-27-03952-s001.zip › Supp_fig4_April_v22026.pdf]

Supp. Figure 5

A

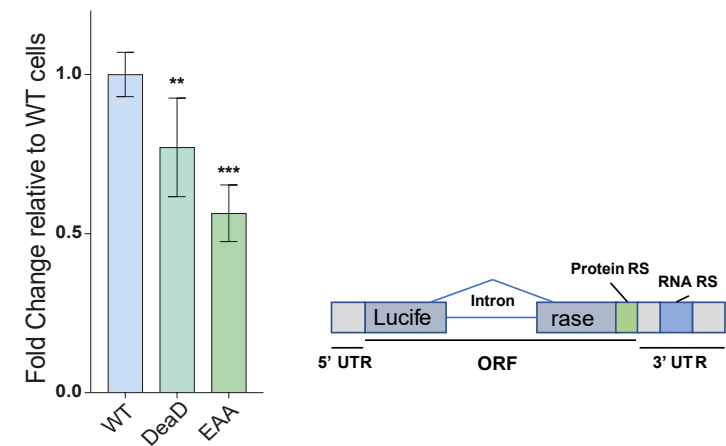

B

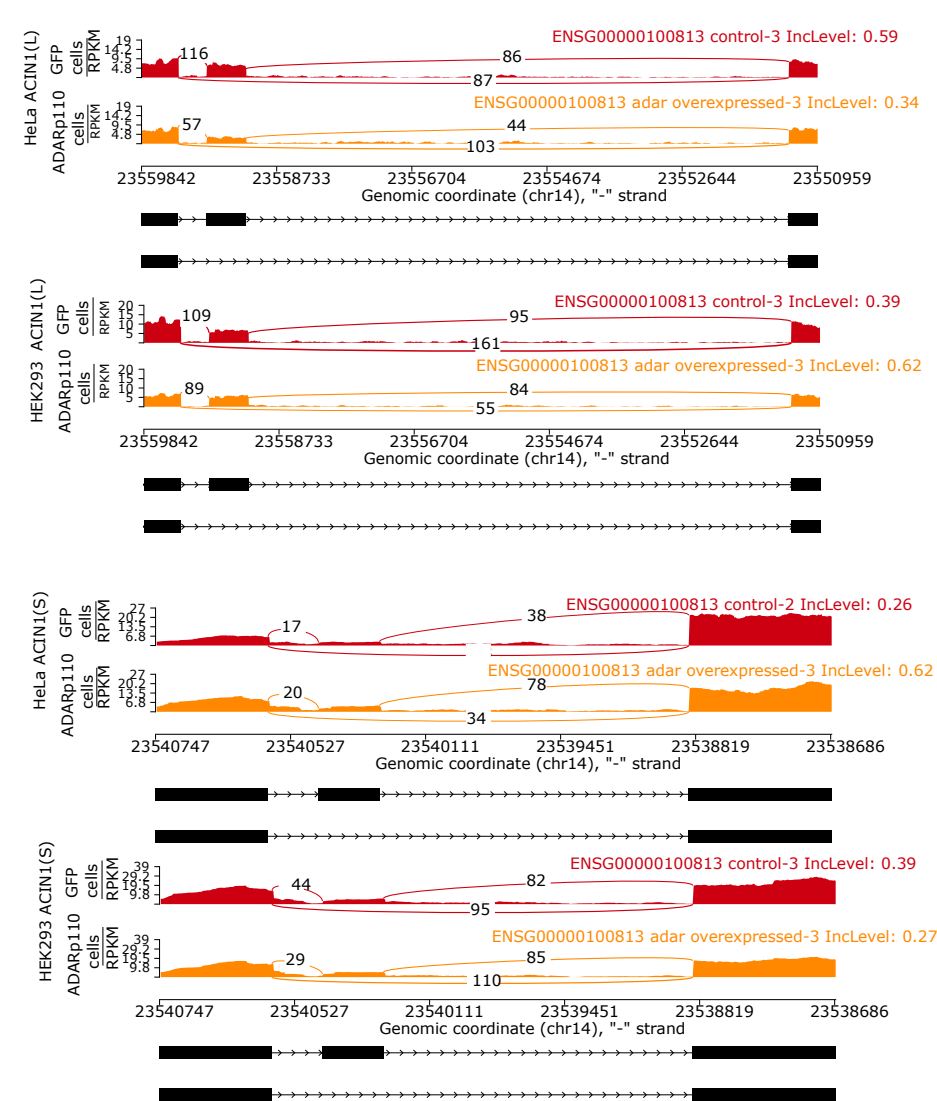

Supplement: Supplementary file 1 [file ijms-27-03952-s001.zip › Supp_fig5_April_v22026.pdf]
